# Human Herpesvirus 6-Associated Miller–Fisher Syndrome in a 5-Year-Old Child: A Case-Based Narrative Review of Pediatric Cases with Infectious Triggers

**DOI:** 10.3390/v18020213

**Published:** 2026-02-06

**Authors:** Ho-Young Song, Kyu Young Chae, Sung-Ha Kim

**Affiliations:** 1 Department of Emergency Medicine, CHA Bundang Medical Center, CHA University School of Medicine, Seongnam 13496, Republic of Korea; shyped85@chamc.co.kr; 2 Department of Pediatrics, CHA Bundang Medical Center, CHA University School of Medicine, Seongnam 13496, Republic of Korea; barnabas@cha.ac.kr

**Keywords:** Miller–Fisher syndrome, pediatrics, neurology, pediatric emergency medicine, human herpesvirus 6, Guillain–Barré syndrome

## Abstract

Background/Objectives: Miller–Fisher syndrome (MFS) is a rare Guillain–Barré variant defined by ophthalmoplegia, ataxia, and areflexia. Pediatric MFS is uncommon, and infectious triggers remain underrecognized. Human herpesvirus 6 (HHV-6) is neurotropic but rarely linked to immune-mediated neuropathies. In this paper, we describe a child with MFS associated with HHV-6 detected in cerebrospinal fluid (CSF) and review reported pediatric infections related to MFS. Methods: A 5-year-old girl presented with acute ophthalmoplegia, ataxia, and diminished reflexes. Neuroimaging, ophthalmologic tests, CSF analyses, and serologic andpolymerase chain reaction (PCR) assays were performed, including multiplex reverse transcription–PCR of cerebrospinal fluid using the BioFire^®^ Meningitis/Encephalitis panel. A literature search was performed on Pubmed to identify pediatric (0–18 years) MFS cases with infectious triggers. Two reviewers independently screened and summarized the literature, and a PRISMA-style flow diagram was used to transparently report the study selection process. Results: HHV-6 DNA was detected via CSF PCR twice, while tests for other pathogens were negative. Anti-GQ1b and related antibodies were negative or borderline. The patient received intravenous immunoglobulin and corticosteroids, with full recovery after one month. Among 20 published pediatric cases (1997–2021), *Campylobacter jejuni* was most frequent, followed by *Mycoplasma pneumoniae* and influenza viruses. Anti-GQ1b IgM positivity and favorable outcomes were commonly reported, including cases managed conservatively. Conclusions: This case raises the hypothesis that HHV-6 may represent a potential post-infectious association in pediatric MFS. The review findings indicate that pediatric MFS generally follows infection, responds well to immunotherapy, and has an excellent prognosis. Viral testing may be considered in selected, hypothesis-generating contexts in atypical or seronegative pediatric MFS presentations.

## 1. Introduction

Miller-Fisher syndrome (MFS) was first described in the 1950s. It is a rare variant of Guillain-Barré syndrome (GBS) [1] with a triad of distinctive characteristics: ophthalmoplegia, areflexia, and ataxia [2].

An acute autoimmune response caused by various preceding infections is considered to be the pathogenesis of MFS [3]. Various bacteria and viruses have been studied due to their association with this syndrome, including *Campylobacter jejuni* and *Haemophilus influenzae* as the most common pathogens [4,5]. Other pathogens such as *Streptococcus pyogenes*, *Staphylococcus aureus*, *Mycoplasma pneumoniae*, *Coxiella burnetii*, *cytomegalovirus*, *Epstein–Barr virus*, *varicella zoster virus*, and *mumps virus* have also been reported [6].

As MFS is even rarer in the pediatric population; its clinical findings and laboratory, including the detection of possible causal infections, are mainly reported in adult patients [7]. Accordingly, this article presents a representative pediatric case of human herpesvirus 6 (HHV-6)-associated MFS and integrates it into a case-based narrative review of pediatric MFS with infectious triggers, focusing on viral etiologies and immune-mediated mechanisms.

## 2. Case Report

A 5-year-old girl presented to our pediatric emergency department with a 4-day history of dizziness, followed by gait disturbance and visual symptoms. Her caregiver reported that the patient had complained about visual disturbances for the past 3 days. Additionally, the caregiver observed that she had been persistently rotating her head to one side, and she began to maintain this posture the day before visiting our department. Further history taking revealed that the patient had recently suffered from gastroenteritis with fever, vomiting, and diarrhea, which had been resolved following treatment at a local outpatient clinic.

**Figure 1:**
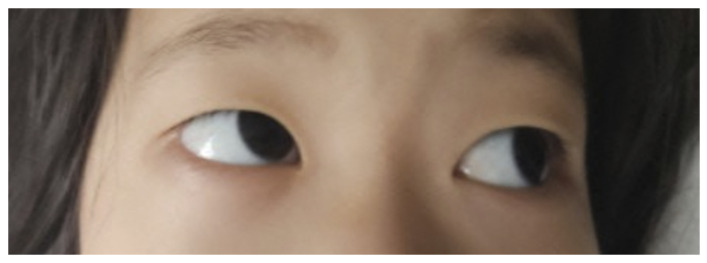
**Ocular findings at presentation.** Ocular alignment and motility findings in a 5-year-old girl with Miller–Fisher syndrome. Right hypertropia and limited ocular movements were observed, consistent with cranial nerve involvement.

On physical examination, the patient’s vital signs were stable. Her height, weight, and body mass index were all within normal ranges for her age. Her general condition and mental status were intact. Her pupil sizes were within normal limits with normal reflexes. However, her extraocular movements were abnormal and suspected to be limited in lateral gaze. Slight ptosis of the right eyelid was also observed. Her eyesight was intact while in her favored position, and when keeping her head slightly rotated to the left side, the patient adopted a compensatory head tilt to alleviate diplopia, which became evident when her head was placed in a neutral position (Figure 1). No meningeal signs were observed, such as neck stiffness, Brudzinski sign, or Kernig sign, and the jolt accentuation test was negative. Although she could walk, the patient’s gait was wobbly, demonstrating gait instability involving both lower extremities, which was suggestive of truncal and proximal lower limb ataxia.

Routine laboratory tests, inflammatory markers, venous blood gas analysis, and chest radiography were unremarkable. A prompt brain computed tomography (CT) scan was conducted, which showed no abnormalities.

After evaluating the non-contrast brain CT scan, lumbar puncture was performed. CSF analysis revealed an elevated white blood cell count (235 cells/µL) with lymphocytic predominance. The CSF glucose and protein levels were 62 mg/dL and 37 mg/dL, respectively. Acid-fast bacilli staining of the CSF was negative. Multiplex reverse transcription–polymerase chain reaction (RT-PCR) testing of the CSF was performed using the BioFire^®^ Meningitis/Encephalitis (ME) panel (bioMérieux, Salt Lake City, UT, USA). This multiplex RT-PCR was positive for human herpesvirus 6 (HHV-6). The results for other bacterial and viral pathogens were negative, including for *Escherichia coli K1*, *Haemophilus influenzae*, *Listeria monocytogenes*, *Neisseria meningitidis*, *Streptococcus agalactiae*, *Streptococcus pneumoniae*, *cytomegalovirus*, *enterovirus*, *herpes simplex virus types 1 and 2*, *human parechovirus*, *varicella zoster virus*, and *Cryptococcus neoformans/gattii*. No organisms were isolated from either the initial or follow-up CSF cultures.

The patient was admitted to the pediatric neurology department with a working diagnosis of meningitis and was started on intravenous third-generation cephalosporin therapy. On the second day of hospitalization, she developed grade I weakness and diffuse hyporeflexia of deep tendon reflexes in both the upper and lower extremities. Given the clinical triad of ophthalmoplegia, areflexia, and ataxia, MFS was diagnosed. Orbital magnetic resonance imaging (MRI) with contrast, ophthalmology consultation, and additional laboratory evaluations were promptly performed. Intravenous immunoglobulin (IVIG) and corticosteroid pulse therapy were initiated thereafter.

Orbital MRI showed no remarkable findings. However, ophthalmologic evaluation demonstrated vertical diplopia and complex ocular misalignment, including right hypertropia, torsional deviation, and variable esotropia/exotropia, consistent with cranial nerve involvement. Extraocular muscle testing suggested impaired function of the right superior oblique and inferior oblique muscles. Our ophthalmologist concluded the presence of obvious misalignment of the visual axis due to strabismus, and the impaired ocular motility was suspected to be secondary to transient inflammatory changes in the central nervous system, resulting in limited extraocular muscle function.

On the third day of hospitalization, additional investigations were conducted, including a second CSF analysis. Serum aldolase was within normal limits, which helped rule out peripheral muscular disorders. Testing for anti-GQ1b IgG antibodies yielded negative results. Testing for immunoglobulin M antibodies against ganglioside GM1 (anti-GM1 IgM) and ganglioside GQ1b (anti-GQ1b IgM) also yielded negative results. The result for immunoglobulin M antibodies against ganglioside GD1b (anti-GD1b IgM) was reported as borderline. A stool test was performed to detect *Campylobacter jejuni*, but it yielded a negative result. The second cerebrospinal fluid test revealed an elevation in white blood cells (388 cells/µL). Cerebrospinal fluid protein was within the normal limits. The multiplex RT-PCR of CSF still showed positive for HHV-6.

The patient began to show clinical improvement starting on the fourth day of hospitalization, after discontinuation of intravenous antibiotics. Antiviral therapy was not administered during the hospital stay. Nerve conduction studies performed on the tenth day of hospitalization yielded normal results. From that day forward, all neurologic symptoms showed progressive improvement, except for persistent mild hyporeflexia. She was discharged the following day. During the 1-month outpatient follow-up, the patient showed complete resolution of ophthalmoplegia, ataxia, and areflexia. Mild fine motor difficulties were noted on rehabilitation assessment, and occupational therapy was continued.

## 3. Materials and Methods

**Figure 2:**
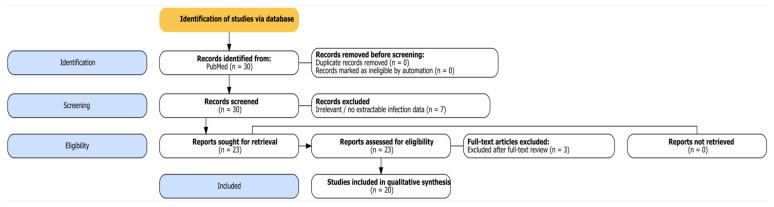
**PRISMA-style flow diagram of study selection.** A total of 30 records were identified, with 7 excluded during abstract screening and 3 after full-text review, resulting in 20 eligible pediatric MFS cases.

We conducted a search on PubMed on 30 September 2025, limited to English, humans, and case reports involving patients aged 0–18 years. The literature search covered all publications indexed in PubMed up to 30 September 2025, without a restriction on publication year. The query combined “Miller–Fisher syndrome” AND (infection OR specific pathogens: *Campylobacter jejuni*, *Haemophilus influenzae*, *Mycoplasma pneumoniae*, *Helicobacter pylori*, *influenza*, *norovirus*, EBV, CMV, HSV, VZV.). Two reviewers independently screened titles/abstracts and full texts extracted predefined variables (age/sex, pathogen and diagnostic method, antiganglioside antibodies, treatment, outcomes), and resolved disagreements via consensus. In total, 30 records were identified, with 7 excluded during the abstract screening stage (irrelevant/no extractable infection data), and 3 during the full-text screening stage. Then, 20 pediatric cases remained for qualitative synthesis (Figure 2). Therefore, we conducted a case-based narrative review of published pediatric (0–18 years) MFS cases with infectious triggers. We used a PRISMA 2020-style flow diagram to transparently report the literature identification and selection process; however, this review was not designed as a formal systematic review.

A structured data extraction sheet was developed to collect the following variables from each article: first author and publication year, age or age range of patients, sex of patients, infectious pathogen identified, diagnostic methods (e.g., CSF PCR, serology, etc.), antiganglioside antibody testing results, treatment modalities (e.g., IVIG, corticosteroids), clinical outcomes. If applicable, data on MFS variants, such as partial triad presentations, were also noted.

## 4. Results

Among the 20 included pediatric MFS cases (1997–2021), *Campylobacter jejuni* was the most frequently reported association, followed by *Mycoplasma pneumoniae* and influenza. Pathogen identification relied mainly on serology, with occasional CSF PCR. Anti-GQ1b IgM was frequently positive; other antiganglioside antibodies were variably detected. IVIG was the predominant therapy, and was sometimes combined with corticosteroids. Recovery was favorable in most cases, with complete resolution typically achieved within weeks to months, while a minority had mild residual diplopia or hyporeflexia during short-term follow-up (Table 1).

## 5. Discussion

Although a rare neuropathy, adult-onset MFS has been well documented. Nonetheless, pediatric cases remain relatively rare with even fewer reports elucidating their potential infectious triggers. To contextualize this case, we conducted a focused literature review of pediatric MFS cases published from 1997 to 2021. *Campylobacter jejuni* was the most frequently associated pathogen, followed *by Mycoplasma pneumoniae* and influenza viruses. In addition to the pathogens identified in this review, few recent pediatric cases temporally associated with SARS-CoV-2 infection have been reported [26]. No consistent pathogen-specific differences in core clinical presentation were identified. Regardless of the implicated pathogen, most patients exhibited the characteristic features of MFS, including ophthalmoplegia with variable ataxia and areflexia. Most diagnoses were supported by serologic testing, and anti-GQ1b antibodies were commonly detected. Treatment typically involved IVIG, with or without corticosteroids. Clinical outcomes were also comparable irrespective of the infectious trigger. Prognosis was favorable in nearly all cases, with full recovery in the majority within weeks to months.

Although HHV-6 is ubiquitous and capable of latency and reactivation, its neurologic relevance has been explored in multiple contexts. Merelli et al. reported significantly higher HHV-6 antibody titers in adult patients with GBS than in controls, suggesting a possible association [27]. In addition, a study on CSF and clinical specimens identified HHV-6 among the more frequently detected herpesviruses in CNS syndromes, underscoring its neurotropic potential [28]. HHV-6 reactivation has been implicated in a range of neurologic disorders, including encephalitis, acute disseminated encephalomyelitis (ADEM), and GBS, suggesting a broader role for HHV-6 in pediatric and adult neuroinflammatory and immune-mediated demyelinating diseases [29]. Experimental and clinical data suggest that HHV-6 infection may influence immune dysregulation through CD4^+^ T-cell tropism, blood–brain barrier penetration, and molecular mimicry mechanisms, thereby amplifying post-infectious autoimmune responses [30]. In this context, the repeated detection of HHV-6 DNA in the CSF of our patient, in the absence of other definite infectious triggers, is compatible with the hypothesis that HHV-6 may represent a potential post-infectious association with MFS. However, alternative explanations, including preceding gastroenteritis and borderline antiganglioside antibody findings, cannot be excluded. While causality cannot be established from a single case, our findings align with emerging evidence that HHV-6 should be considered in seronegative or atypical pediatric MFS presentations, particularly when CSF pleocytosis or central nervous system involvement is observed.

Pediatric MFS remains a diagnosis that requires high clinical suspicion, especially in patients with acute ophthalmoplegia and ataxia. The literature consistently describes pediatric MFS as a post-infectious condition with favorable outcomes, emphasizing the importance of early recognition and appropriate supportive or immunomodulatory management [31].

Anti-GQ1b antibodies were reported in the majority of pediatric cases, although the immunoglobulin subclass varied across reports, including IgG-only, IgM-only, combined IgG/IgM positivity, and cases without subclass specification. A smaller number of patients exhibited additional antiganglioside antibodies, such as anti-GT1a or anti-GD1b, which have been reported less consistently. Management strategies varied widely across cases, ranging from IVIG therapy, with or without corticosteroids, to supportive care alone. IVIG was the most frequently reported therapeutic intervention. Overall outcomes were favorable in most reported patients, with 15 of 19 achieving complete recovery within weeks to months. A minority experienced mild residual symptoms, such as diplopia or hyporeflexia, and follow-up was incomplete in one case. Altogether, these findings suggest that pediatric MFS, although rare, is generally associated with a favorable clinical course [32].

This study has several important limitations. First, as a single case report, it presents low-level evidence and does not allow causal inference regarding the relationship between HHV-6 and MFS. Interpretation of HHV-6 detection in cerebrospinal fluid requires caution, as HHV-6 is ubiquitous and capable of latency and reactivation. Furthermore, chromosomally integrated HHV-6 (ciHHV-6), which can result in persistently detectable viral DNA independent of acute infection, cannot be excluded [33]. In addition, alternative post-infectious factors, including the patient’s preceding gastroenteritis and borderline antiganglioside antibody findings, cannot be fully ignored. Second, the diagnostic evaluation had inherent constraints. Although anti-GQ1b antibodies are characteristic of MFS, they are absent in a substantial subset of patients, and the borderline anti-GD1b result in this case cannot be conclusively interpreted. Nerve conduction studies were performed later in the disease course and yielded normal results, which may have limited their diagnostic contribution. Third, the literature component of this work was designed as a case-based narrative review to contextualize a rare pediatric presentation rather than as a systematic review. Therefore, formal risk-of-bias assessment, evidence grading, or quantitative synthesis was not performed. Because the available pediatric MFS literature predominantly consists of isolated case reports with substantial clinical and methodological heterogeneity, inferential comparisons, heterogeneity assessments, or sensitivity analyses were not feasible. Accordingly, findings from the literature review should be interpreted as descriptive and hypothesis-generating rather than practice-changing.

To our knowledge, this is the first reported pediatric case of MFS in which HHV-6 was detected in cerebrospinal fluid in the absence of other identified pathogens. While causality cannot be definitively established from a single case, this report highlights the need to consider HHV-6 in the differential diagnosis of post-infectious neurologic syndromes such as MFS. Further research is warranted to investigate the pathogenic potential of HHV-6 in pediatric MFS and to determine whether routine screening for this virus in suspected cases is justified.

## Figures and Tables

**Table 1:** **Summary of reported pediatric cases of Miller–Fisher syndrome associated with infectious pathogens**.

First Author and Publication Year	Age	Sex	Infectious Pathogen Identified	Diagnostic Method	Antiganglioside Antibody Testing Results	Treatment Modalities	Clinical Outcomes
Spyromitrou-Xioufi et al., 2021 [8]	6	M	VZV	Serologic test	Anti-GQ1b antibody positive (subclass not specified)	IVIG + High-dose methylprednisolone	Improved after treatment; full recovery observed during 4-month follow-up.
Communal et al., 2016 [9]	14	M	EBV	Serologic test	Anti-GQ1b IgG positive	IVIG	Improved after treatment; full recovery observed during 6-month follow-up.
Wawrzusin et al., 2021 [10]	16	F	*Helicobacter pylori*	Serologic test	Anti-GQ1b antibody positive	IVIG	Improved after treatment; not fully recovered during 1-month follow-up.
Moustaki et al., 2012 [11]	10	M	*Enterovirus*	PCR (CSF)	Anti-GQ1b antibody positive	IVIG	Improved after treatment; full recovery observed during 4-month follow-up.
Costiniuk et al., 2011 [12]	1	M	*Influenza A*	PCR (Nasopharyngeal aspirate)	Not tested	IVIG	Improved after treatment; mild areflexia during 4-month follow-up.
Dutta et al., 2018 [13]	5	F	*Chikungunya virus*	Serologic test	Not reported	Oral steroid	Follow-up information lost.
Guisset et al., 2016 [14]	6	M	*Campylobacter jejuni*	Serologic test	Anti-GQ1b IgM, anti-GQ1b IgG, anti-GT1a IgM, anti-GT1a IgG positive	No specific Tx.	Full recovery observed during 7-week follow-up.
Blasetti et al., 2007 [15]	10	F	*Escherichia coli*	Urine culture	Nothing positive	IVIG	Improved after treatment; full recovery observed during 2-month follow-up.
Akinci et al., 2010 [16]	13	F	*Mycoplasma pneumoniae*	Serologic test	Nothing positive	IVIG	Improved after treatment; full recovery observed during 4-week follow-up.
Morgan et al., 2016 [17]	18	M	*Influenza B*	Serologic test	Nothing positive	Conservative Tx. only	Full recovery observed during 5-month follow-up.
Lee, 2012 [18]	13	M	*Campylobacter jejuni*	Serologic test	Nothing positive	IVIG	Improved after treatment; mild diplopia and knee jerk left during 3-month follow-up.
Steer et al., 2006 [19]	11	M	*Mycoplasma pneumoniae*	Serologic test	Anti-GQ1b IgG positive	IVIG	Improved after treatment; full recovery observed during 4-month follow-up.
Davis et al., 2008 [20]	18	M	*Campylobacter jejuni*	Stool culture	Not reported	IVIG	Improved after treatment; full recovery on day 10.
Matsumoto et al., 2002 [21]	4	M	*Influenza B*	Serologic test	Anti-GQ1b IgG positive	IVIG	Improved after treatment; full recovery on day 8.
Kuroki et al., 2001 [22]	11	M	*Campylobacter jejuni*	Serologic test	Anti-GQ1b IgG positive	Conservative Tx. only	Full recovery observed during 8-month follow-up.
Kuroki et al., 2001 [22]	14	M	*Campylobacter jejuni*	Serologic test	Anti-GQ1b IgG, anti-GQ1b IgM, anti-GM1 IgM, anti-GD1a IgM, anti-GD1b IgM positive	Conservative Tx. only	Full recovery observed during 2-month follow-up.
Kuroki et al., 2001 [22]	3	M	*Campylobacter jejuni*	Serologic test	Anti-GQ1b IgM positive	Conservative Tx. only	Full recovery observed during 1-month follow-up.
Matsubara et al., 1997 [23]	6	F	*Campylobacter jejuni*, *Herpes simplex virus*	Serologic test	Anti-GQ1b IgG, anti-GQ1b IgM, anti-GT1b IgG positive	Acyclovir + High-dose methylprednisolone	No effect from treatment, self-limited; full recovery observed during 3-month follow-up.
Mewasingh et al. [24]	9	F	*Campylobacter jejuni*	Serologic test	Anti-GQ1b antibody positive (subclass not specified)	IVIG	Full recovery observed during 1-month follow-up.
Hsueh et al., 2004 [25]	6	F	*Mycoplasma pneumoniae*	Serologic test	Not reported	IVIG	Improved after treatment; full recovery observed during 4-week follow-up.

Abbreviations: IVIG = intravenous immunoglobulin; Tx = treatment; PCR = polymerase chain reaction; CSF = cerebrospinal fluid; VZV = *varicella zoster virus*; EBV = *Epstein–Barr virus*.

## Data Availability

Dataset available upon request from the authors.

## Abbreviations

The following abbreviations are used in this manuscript: MFS, Miller–Fisher syndrome; GBS, Guillain–Barré syndrome; HHV-6, human herpesvirus 6; CSF, cerebrospinal fluid; PCR, polymerase chain reaction; IVIG, intravenous immunoglobulin; EBV, Epstein–Barr virus; CMV, cytomegalovirus; HSV, herpes simplex virus; VZV, varicella zoster virus; MRI, magnetic resonance imaging; AFB, acid-fast bacilli; Tx, treatment; PRISMA, Preferred Reporting Items for Systematic Reviews and Meta-Analyses.
